# ASK120067 potently suppresses B-cell or T-cell malignancies *in vitro* and *in vivo* by inhibiting BTK and ITK

**DOI:** 10.3389/fphar.2022.1071114

**Published:** 2022-12-15

**Authors:** Peiran Song, Gang Bai, Shingpan Chan, Tao Zhang, Linjiang Tong, Yi Su, Yanyan Shen, Yi Chen, Yingqiang Liu, Mengzhen Lai, Yi Ning, Haotian Tang, Yan Fang, Yi Chen, Ke Ding, Jian Ding, Hua Xie

**Affiliations:** ^1^ Zhongshan Institute for Drug Discovery, Shanghai Institute of Materia Medica, Chinese Academy of Sciences, Zhongshan, China; ^2^ Division of Antitumor Pharmacology, State Key Laboratory of Drug Research, Shanghai Institute of Materia Medica, Chinese Academy of Sciences, Shanghai, China; ^3^ College of Pharmacy, Jinan University, Guangzhou, China; ^4^ Department of Pharmacology, School of Pharmacy, Fudan University, Shanghai, China; ^5^ Hangzhou Institute for Advanced Study, University of Chinese Academy of Sciences, Hangzhou, China

**Keywords:** Bruton’s tyrosine kinase, interleukin-2-inducible T cell kinase, ASK120067, B-cell lymphoma, T-cell leukemia

## Abstract

Hyperactivation of Bruton’s tyrosine kinase (BTK) or interleukin-2-inducible T cell kinase (ITK) has been attributed to the pathogenesis of B-cell lymphoma or T-cell leukemia, respectively, which suggests that Bruton’s tyrosine kinase and interleukin-2-inducible T cell kinase are critical targets for the treatment of hematological malignancies. We identified a novel third-generation epidermal growth factor receptor (EGFR) inhibitor, ASK120067 (limertinib) in our previous research, which has been applied as a new drug application against non-small cell lung cancer in China. In this work, we found that ASK120067 displayed potent *in vitro* inhibitory efficacy against Bruton’s tyrosine kinase protein and interleukin-2-inducible T cell kinase protein *via* covalent binding. In cell-based assays, ASK120067 dose-dependently suppressed Bruton’s tyrosine kinase phosphorylation and exhibited anti-proliferation potency by inducing apoptosis in numerous B-lymphoma cells. Meanwhile, it caused growth arrest and induced the apoptosis of T-cell leukemia cells by attenuating interleukin-2-inducible T cell kinase activation. Oral administration of ASK120067 led to significant tumor regression in B-cell lymphoma and T-cell leukemia xenograft models by weakening Bruton’s tyrosine kinase and interleukin-2-inducible T cell kinase signaling, respectively. Taken together, our studies demonstrated that ASK120067 exerted preclinical anti-tumor activities against B-/T-cell malignancy by targeting BTK/ITK.

## Introduction

Bruton’s tyrosine kinase (BTK) is a key component of B-cell receptor (BCR) signaling. BTK regulates B-cell survival, and also plays a critical part in signaling of Toll-like receptor and chemokine receptor, which can regulate cell migration and the tumor microenvironment (Liang et al., 2018). Dysregulation of BTK may induce a wide array of diseases involving activation of B-cell, dendritic cells, or macrophages, such as B-cell malignancies, asthma and rheumatoid arthritis (Zarrin et al., 2021). These features of BTK make it a very attractive drug target in the treatment of B-cell malignancies and autoimmune diseases (Pal Singh et al., 2018; Ringheim et al., 2021).

The first-in-class BTK inhibitor ibrutinib has been approved by the United States Food and Drug Administration for the treatment of mantle cell lymphoma (MCL), chronic lymphocytic leukemia (CLL), Waldenström’s macroglobulinemia (WM), marginal zone lymphoma (MZL) and chronic graft versus host disease (cGVHD) (Akinleye et al., 2013; Parmar et al., 2014; Wen et al., 2021). Ibrutinib has been shown to abrogate BTK activation irreversibly by inhibiting its autophosphorylation at tyrosine residue 223. This action leads to inhibition of downstream phosphorylation of essential cell-survival pathways (e.g., phospholipase-Cγ2, PLCγ2) to achieve an objective therapeutic effect (Kim, 2019; Gaballa and Pinilla-Ibarz, 2021).

As a T-cell dominant member of the TEC kinase family, interleukin-2-inducible T cell kinase (ITK) drives proximal T-cell-receptor signaling (Mammadli et al., 2020; Solouki et al., 2020; Wallace et al., 2021). ITK is stimulated upon ligand of T -cell receptors in T cells, followed by initiating phospholipase-Cγ1 (PLCγ1) for cascade signal transduction (Conley et al., 2020; Lechner et al., 2020; Lowe et al., 2022). ITK is activated aberrantly and overexpressed in T-cell acute lymphoblastic leukemia and Sezary syndrome/cutaneous T-cell lymphoma, and has been shown to be a key signaling motif in these cells (Guo et al., 2012; Litvinov et al., 2017). ITK inhibitors could be treatment for T-cell lymphoma that is difficult to manage. Multiple chemical analogs have been reported, but selective inhibitors of ITK approved for the treatment of T-cell lymphoma are lacking. Therefore, discovering clinically efficacious ITK inhibitors is very important (Sahu and August 2009;Litvinov et al., 2017; Lechner et al., 2020).

The potent inhibition of ITK by ibrutinib is due to the significant structural homology between BTK and ITK. Ibrutinib treatment has been demonstrated to: i) increase the *in vivo* persistence of activated cluster of differentiation CD4^+^ and CD8^+^ T cells; ii) diminish expression of immunosuppressive molecules in CLL cells; iii) exhibit robust immunomodulatory effects (Dubovsky et al., 2013; Long et al., 2017).

Previously, we developed a novel and potent third-generation epidermal growth factor receptor (EGFR) tyrosine kinase inhibitor ASK120067 (limertinib). ASK120067 inhibited EGFR tyrosine kinase inhibitor-sensitizing mutations and T790M-resistant mutations while sparing the activity of wild-type EGFR (Zhang et al., 2020). ASK120067 showed considerable clinical benefits and safety in patients with EGFR T790M mutant non-small cell lung cancer (NSCLC) in clinical trial (NCT04143607). A new drug application has been submitted for ASK120067 in China. ASK120067 could inhibit mutant EGFR by binding covalently to the kinase at cysteine 797 (Cys797) in the adenosine triphosphate (ATP)-binding domain. BTK and ITK share a highly conserved cysteine molecule with EGFR Cys797. Also, EGFR inhibitors have been reported to possess BTK inhibitory activity (Grabinski and Ewald, 2014; Guo et al., 2014; Wang et al., 2016; Liang et al., 2017). Thus, we investigated if ASK120067 could inhibit BTK and ITK directly, and the potential therapeutic effects of ASK120067 against lymphoma and leukemia.

Herein, we identified that the third-generation EGFR inhibitor ASK120067 exhibited potency against B-cell lymphoma and T-cell leukemia by targeting BTK and ITK, respectively. These evaluations might lead to expansion of the clinical indications of ASK120067.

## Materials and methods

### Kinase activity assay

An enzyme-linked immunosorbent assay (ELISA) was applied to evaluate the kinase activity of BTK and ITK. BTK protein (#14-552) and ITK protein (#14-660) were purchased from MilliporeSigma (Burlington, MA, United States). Experiments were undertaken according to standard ELISA procedures (Xue et al., 2018). The absorbance value at 490 nm of each well was used as the original result. Half-maximal inhibitory concentration (IC_50_) values were calculated from the inhibition curves from three independent experiments.

### Molecular modeling

Published structures of the BTK protein (PDB ID: 5P9K) and ITK protein (PDB ID:3QGW) were used for modeling of the potential binding modes of ASK120067 with CovDock, a covalent docking program from Schrödinger (www.schrodinger.com/). Ligands were prepared by the LigPrep module within Schrödinger. Protein structures were optimized by the Protein Preparation Wizard module in the Maestro (www.schrodinger.com/products/maestro/). Choosing cysteine (Cys481 to BTK or Cys442 to ITK) as the reactive residue of proteins, the centroid of ligand (CNX-774 to BTK or L7a to ITK) was confined to the enclosing box, and the reaction type was set as a Michael Addition. Other parameters were left at default settings.

### Cell culture and agents

Ramos and Mino cells were cultured in RPMI 1640 (#C11875500BT, Gibco) plus 10% fetal bovine serum (FBS) (#10099-141, Gibco). SU-DHL-6, Jurkat and Molt-4 cells were cultured in RPMI 1640 plus 10% FBS but also containing glucose (#20180109, Sinopharm Group) and sodium pyruvate (#20180109, Sinopharm Group). All cell types above were cultured in an incubator in an atmosphere of 5% CO_2_ at 37°C. Cell lines were obtained from American Type Culture Collection (Manassas, VA, United States) except for Mino cells (Cobioer Biotechnology, Nanjing, China).

ASK120067 was provided by Aosaikang Pharmaceuticals (Nanjing, China). Ibrutinib (#S2680) was purchased from Selleck Chemicals (Houston, TX, United States).

### Western blotting

Western blotting was conducted using a conventional method. Briefly, cells were lysed in 1× lysis buffer and boiled at 100°C for 15 min. Cell lysis solution was loaded onto gels and sodium dodecyl sulfate–polyacrylamide gel electrophoresis carried out. Proteins were transferred onto nitrocellulose membranes. After blockade with 2% bovine serum albumin (BSA) for 1 h at room temperature, nitrocellulose membranes were incubated with antibodies overnight at 4°C: ITK (#2380), phosphorylated (p)-ITK (#5082), BTK (#8547), p-BTK (#87141) (all from Cell Signaling Technology, Danvers, MA, United States), or β-actin (#60008-1-lg, Proteintech). Anti-immunoglobulin (Ig)M (#9023-01, Southern Biotech, Birmingham, AL, United States) was utilized for the initiation of BTK signaling. Anti-CD3 (#300465)/anti-CD28 (#302923) (Biolegend, San Diego, CA) were used to stimulate the ITK signaling pathway.

### Cell-proliferation assay

Cell proliferation was determined using the 3-(4,5-Dimethylthiazol-2-yl)-2,5-diphenyltetrazolium bromide (MTT) assay or Cell Counting Kit-8 (CCK-8) assay (Vazyme Biotech). First, cells were seeded in 96-well plates (1.2 × 10^4^ cells/well) for 2 h and exposed to various concentrations of ASK120067 or ibrutinib for 72 h.

We added 20 μl of MTT (5 mg/ml) to each well. Four-hours later, we added sodium dodecyl sulfate lysis buffer (100 μl). The absorbance of each well was recorded at 570 nm using a multi-well spectrophotometer (SPECTRAmax™ PLUS 384; Molecular Devices, Silicon Valley, CA, United States).

Each well was labeled with CCK-8 solution and the absorbance was measured using a multi-well spectrophotometer (SPECTRAmax PLUS 384) at 450 nm. The anti-proliferative activity of ASK120067 and ibrutinib was calculated as [1−(Absorbance_treated_/Absorbance_control_)] × 100%. Each experiment was repeated at least twice.

### Apoptosis

Cancer cells were treated with dimethyl sulfoxide (control) or ASK120067 at the indicated concentrations. Cells undergoing apoptosis were identified using an annexin V-FITC/PI Apoptosis Detection Kit (#A211-02, Vazyme) following the manufacturer’s instructions.

### Anti-tumor activity *in vivo*

All animal experiments complied with the ethical guidelines of animal care set by our institution.

SU-DHL-6 cells or Molt-4 cells were injected into the right flank of each mouse at 5×10^6^ cells per mouse. Mice were assigned randomly into a vehicle group (*n* = 12/group) and treatment groups (*n* = 6/group). The vehicle group was given 1% Tween 80. Treatment groups received ASK120067 (100 or 25 mg/kg, p.o., once daily) for 21 days.

Tumor volume (TV) was calculated using the formula: TV = 0.5 × [length (mm) × width^2^ (mm^2^)]. Relative tumor volume (RTV) was calculated using the formula: RTV = V_t_/V_0_, where V_t_ is the tumor volume on each day, and V_0_ is the tumor volume at the beginning of treatment. Tumor growth inhibition (TGI) was calculated using the formula: TGI% = [1 − (TV_t_ − TV_0_)/(CV_t_ − CV_0_)] × 100%.

### Statistical analysis

The two-tailed Student’s *t*-test was used to determine the significance between data for the control group vs. treatment group. *P* < 0.05 was considered significant. Data were presented as mean ± standard deviation (SD).

## Results

### ASK120067 inhibited the kinase activity of BTK and ITK

**FIGURE 1:**
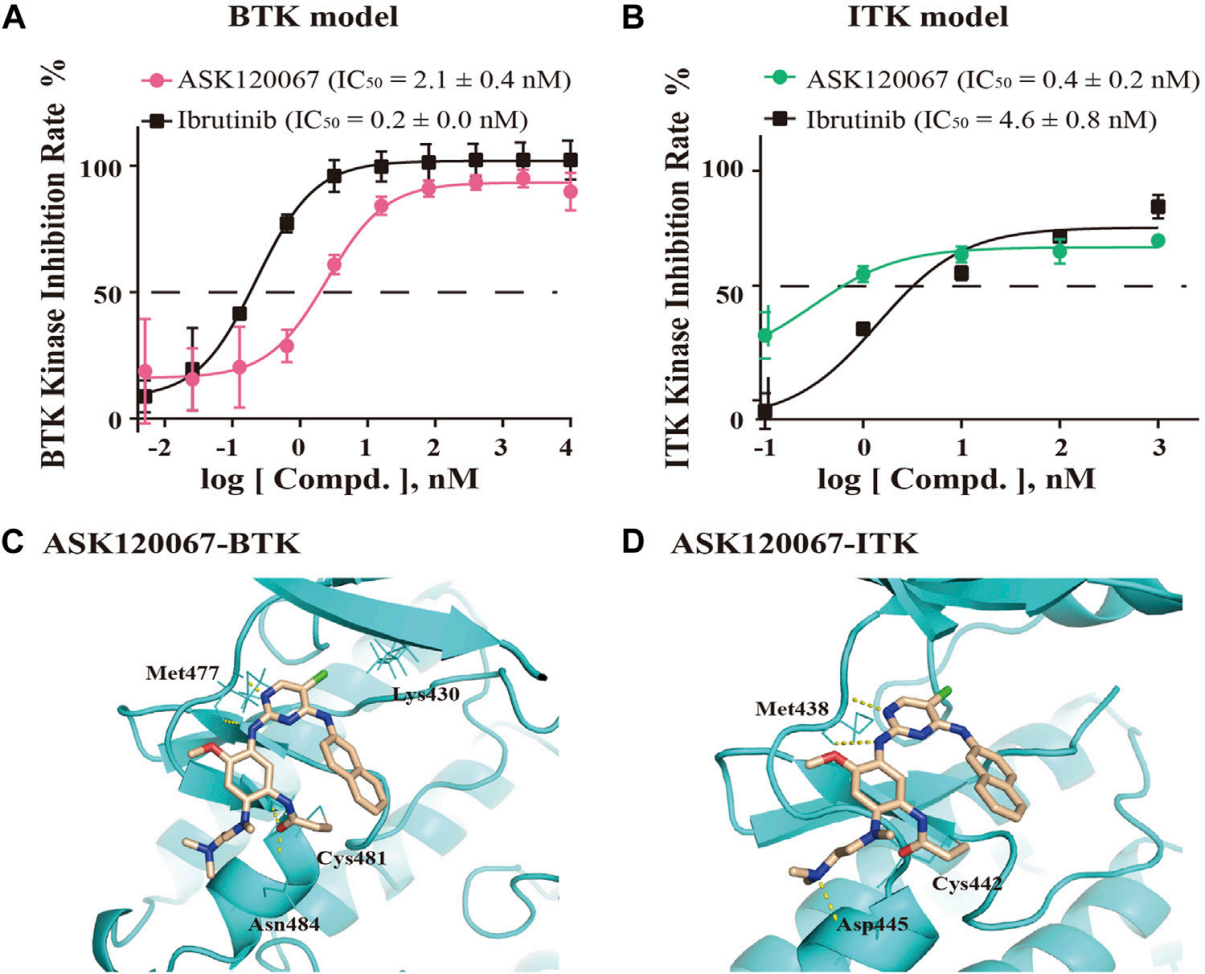
ASK120067 was bound covalently to BTK/ITK and inhibited the activity of their kinases. The kinase-inhibitory activities of ASK120067 or ibrutinib against BTK **(A)** and ITK **(B)** were measured using ELISAs. Data are the mean ± SD and experiments were repeated thrice independently. **(C)** The binding conformation of ASK120067 to the kinase domain of human BTK. ASK120067 formed two hydrogen bonds to the residue Met477, and a covalent bond to the conserved residue Cys481 in the ATP-binding pocket. **(D)** Molecular modeling of the binding mode of ASK120067 with the kinase of ITK. ASK120067 formed two hydrogen bonds to the residue Met438 and a covalent bond to the conserved residue C442.

We undertook ELISAs to evaluate the *in vitro* kinase activity of BTK protein and ITK protein incubated with different concentrations of ASK120067. ASK120067 potently and dose-dependently suppressed the kinase activities of BTK and ITK (Figures 1A, B), with IC_50_s at 2.1 nM or 0.4 nM, respectively, which were similar to those of ibrutinib (IC_50_ = 0.2 nM for BTK and 4.6 nM for ITK). Hence, ASK120067 appeared to be a potent inhibitor of BTK and ITK.

Next, we conducted molecular modeling to simulate the binding between ASK120067 and BTK or ITK to illustrate the underlying structure-activity relationship (SAR). Structural modeling of ASK120067 binding to BTK (PDB ID: 5P9K) (Figure 1C) showed that the 2,4-disubstituted pyrimidine of ASK120067 adopted a U-shaped molecular scaffold, and in the hinge region of BTK protein was formed two hydrogen bonds at the Met477 position. Moreover, ASK120067 formed a covalent bond with Cys481 located in the ATP-binding site of BTK, and the amine moiety extended to the solvent area. Molecular modeling of the interaction between ASK120067 and ITK (PDB ID: 3QGW) was similar to that observed between ASK120067 and BTK, with a U-shaped binding conformation at the ATP pocket. Similarly, the 2-aminopyrimidine core formed two hydrogen bonds to the hinge-residue Met438, and acrylamide formed a covalent bond with Cys442 (Figure 1D). These results demonstrated that ASK120067 was a potent inhibitor of the kinases of BTK and ITK through an irreversible binding mode.

### ASK120067 blocked BTK/ITK activation in malignant cell lines

We then further examine the cellular activity of ASK120067 on the activation of BTK or ITK. We measured the phosphorylation of BTK and ITK in B-cell lymphoma or T-cell leukemia cell lines treated with increasing doses of ASK120067 using western blotting.

**FIGURE 2:**
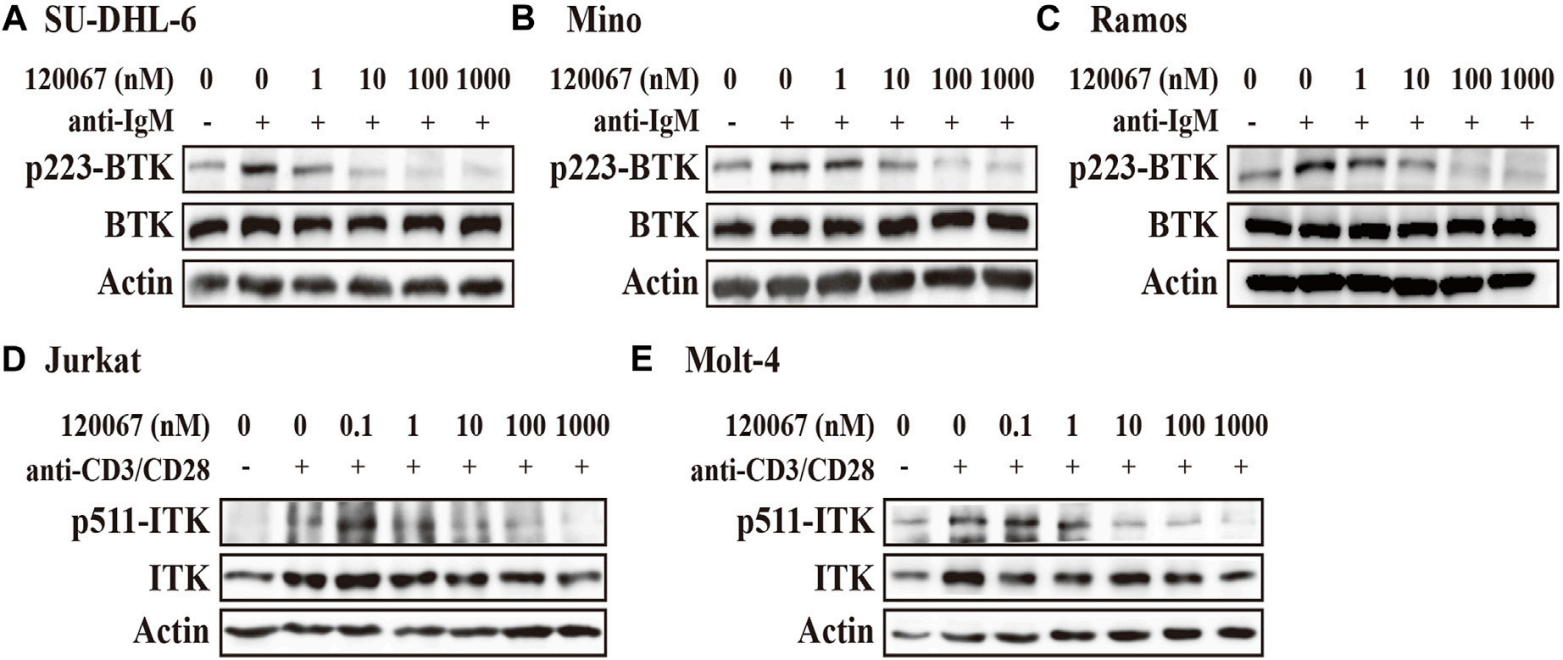
ASK120067 reduced the phosphorylation of BTK or ITK in B-lymphoma or T-cell leukemia cell lines, respectively. The B-lymphoma cell lines SU-DHL-6 **(A),** Mino **(B)**, and Ramos **(C)** were co-incubated with ASK120067 for 2 h and western blotting was done to measure the inhibitory activity of ASK120067 upon BTK activation. The T-cell leukemia cell lines Jurkat **(D)** and Molt-4 **(E)** were exposed to ASK120067 treatment for 2 h, and then ITK phosphorylation were detected by western blotting.

The results showed that ASK120067 dose-dependently suppressed anti-IgM stimulated BTK phosphorylation at Y223 in the diffuse large B-cell lymphoma (DLBCL) cell line SU-DHL-6 (Figure 2A). Inhibitory activity started at a concentration of the compound as low as 1 nM, and was enhanced dramatically at higher concentrations. Inhibition of p-BTK by ASK120067 was also observed in two other B-cell lymphoma cells: the mantle cell lymphoma (MCL) cell line Mino (Figure 2B) and Burkitt’s lymphoma cell line Ramos (Figure 2C).

Meanwhile, ASK120067 also exhibited potently anti-ITK activity in T-cell leukemia. That is, ASK120067 (1 nM–1,000 nM) inhibited the phosphorylation of ITK significantly in a dose-dependent manner in the acute T lymphocyte leukemia (T-ALL) cell lines Jurkat (Figure 2D) and Molt-4 (Figure 2E) cells. These results indicated that ASK120067 could attenuate the activity of BTK or ITK.

### ASK120067 inhibited proliferation and induced apoptosis in B-cell lymphoma cell lines

**FIGURE 3:**
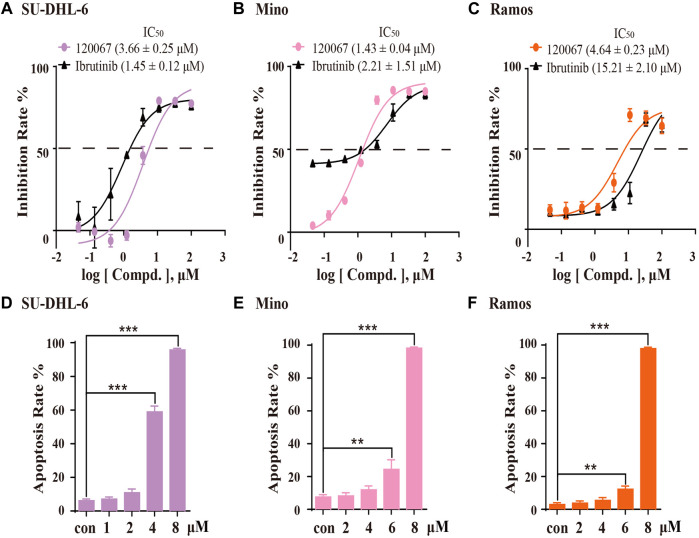
ASK120067 exhibited anti-proliferation and apoptosis-inducing activities in B-lymphoma cells. SU-DHL-6 **(A)**, Mino **(B)**, and Ramos **(C)** cells were incubated with various concentrations of ASK120067 or ibrutinib for 72 h, and then cell viability was measured using the MTT assay. Experiments were undertaken at least twice independently, and data are the mean ± SD. SU-DHL-6 **(D)**, Mino **(E)**, and Ramos **(F)** cell lines were seeded in six-well plates at 2.5 × 10^5^ cells/well and treated with ASK120067 at indicated concentrations, and then apoptosis was analyzed by annexin V/propidium iodide and flow cytometry. Experiments were conducted thrice independently and a significant difference between single-agent groups vs. the control group was determined by the Student’s *t*-test. **p < 0.01; ***p < 0.001.

Based on the inhibitory potency of ASK120067 against BTK, we then evaluated the *in vitro* anti-proliferation effect of ASK120067 against B-cell malignancies. Growth inhibition rate in SU-DHL-6, Mino and Ramos cells were evaluated using the MTT assay after cells had been treated with ASK120067 or ibrutinib for 72 h. ASK120067 inhibited proliferation of these B-cell lymphoma lines effectively in a dose-dependent manner, with IC_50_ values (μM) of 3.66, 1.43 and 4.64 in SU-DHL-6, Mino and Ramos cells (Figures 3A–C), respectively; similar inhibitory effects were observed upon ibrutinib treatment.

Then, we investigated the apoptosis-inducing activity of ASK120067 by flow cytometry. In SU-DHL-6 cells (Figure 3D), ASK120067 triggered significant apoptosis of cells, with percent apoptosis of 58.93% and 95.72% at dose of 4 μM and 8 μM, respectively, compared with 6.11% in untreated (control) cells. Similar apoptosis-inducing activities of ASK120067 were observed in Mino (Figure 3E) and Ramos cell lines (Figure 3F). These results indicated that ASK120067 blocked the growth of B-cell lymphoma cells by triggering cell apoptosis.

### ASK120067 suppressed cell growth and caused the apoptosis of T-cell leukemia cell lines

**FIGURE 4:**
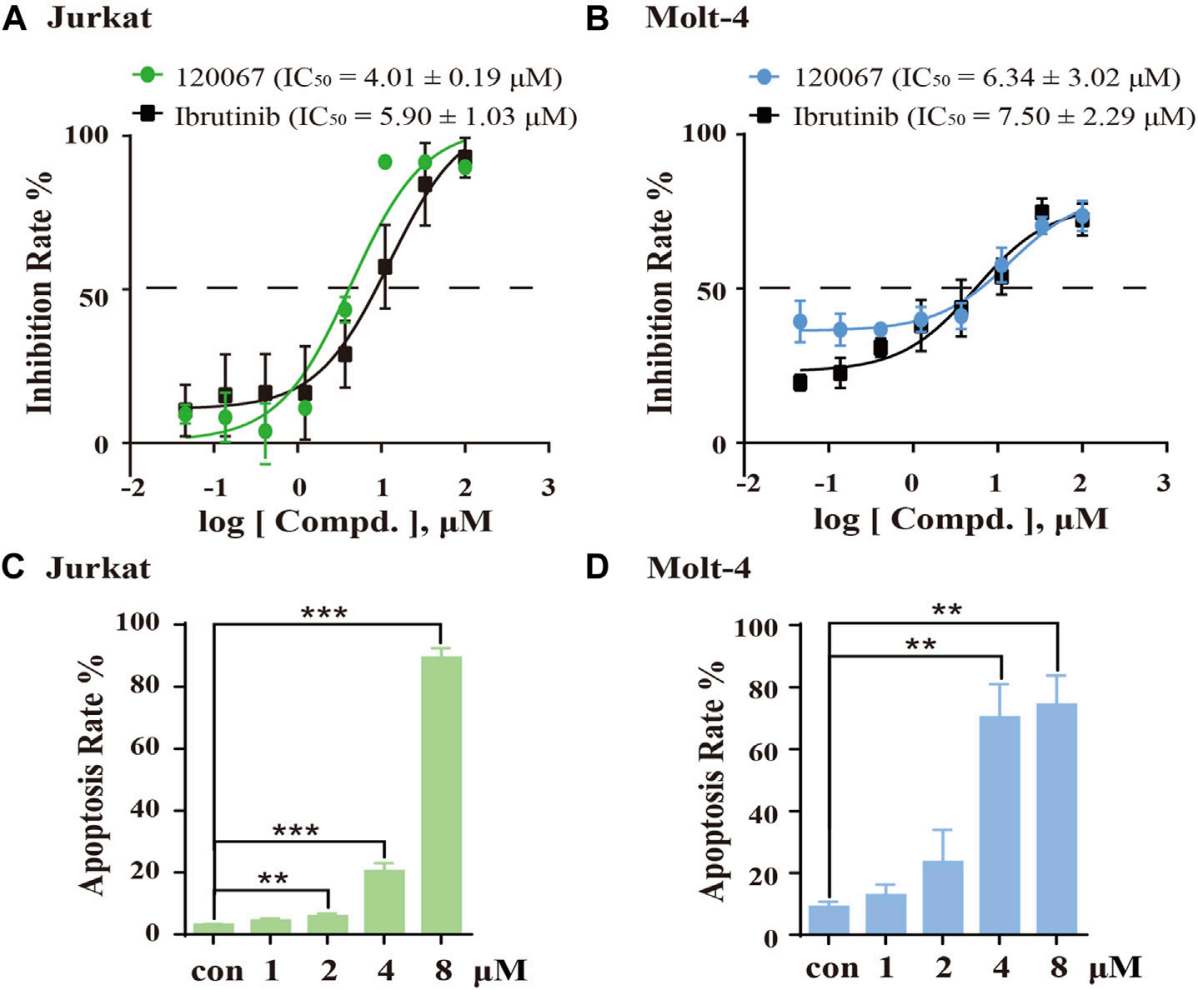
ASK120067 inhibited the growth and induced apoptosis in T-cell leukemia. The CCK-8 assay was used to measure the proliferation of Jurkat **(A)** and Molt-4 **(B)** cells after exposure to various concentrations of ASK120067 or ibrutinib for 72 h. Experiments were undertaken at least twice independently, and results are the mean ± SD. Jurkat **(C)** and Molt-4 **(D)** cells were cultured with different doses of ASK120067 for 48 h and then stained with annexin V/propidium iodide. Percent apoptosis was analyzed by flow cytometry. Experiments were conducted thrice independently and a significant difference between single-agent groups vs. the control group was determined by the Student’s *t*-test. **p < 0.01; ***p < 0.001.

As considerable ITK-inhibition activity of ASK120067 was observed, we explored the anti-proliferative activity of ASK120067 against the T-ALL cell lines Jurkat and Molt-4, which are widely used to evaluate the activity of ITK inhibitors (Guo et al., 2012; Mamand et al., 2018). ASK120067 potently inhibited cell growth in Jurkat cells and Molt-4 cells with IC_50_ values of 4.01 μM and 6.34 μM, respectively (Figures 4A, B).

Subsequently, we examined the effects of ASK120067 on the apoptosis of these cells. Treatment of Jurkat cells and Molt-4 cells with ASK120067 led to a concentration-dependent increase in their apoptosis. Approximately 20.53% of Jurkat cells and 70.25% of Molt-4 cells underwent apoptosis following ASK120067 treatment (4 μM). When the ASK120067 concentration was added to 8 μM, 89.27% of Jurkat cells and 74.41% of Molt-4 cells underwent apoptosis (Figures 4C, D). These results suggested that the anti-tumor effects of ASK120067 against T-cell leukemia could be attributed to inducing the apoptosis of tumor cells.

### ASK120067 demonstrated *in vivo* anti-tumor activity in xenograft model of B- and T-cell malignancies

We then established a B-cell lymphoma xenograft model using SU-DHL-6 cells, and a T-cell leukemia xenograft model using Molt-4 cells to determine the *in vivo* anti-tumor activity of ASK120067.

**FIGURE 5:**
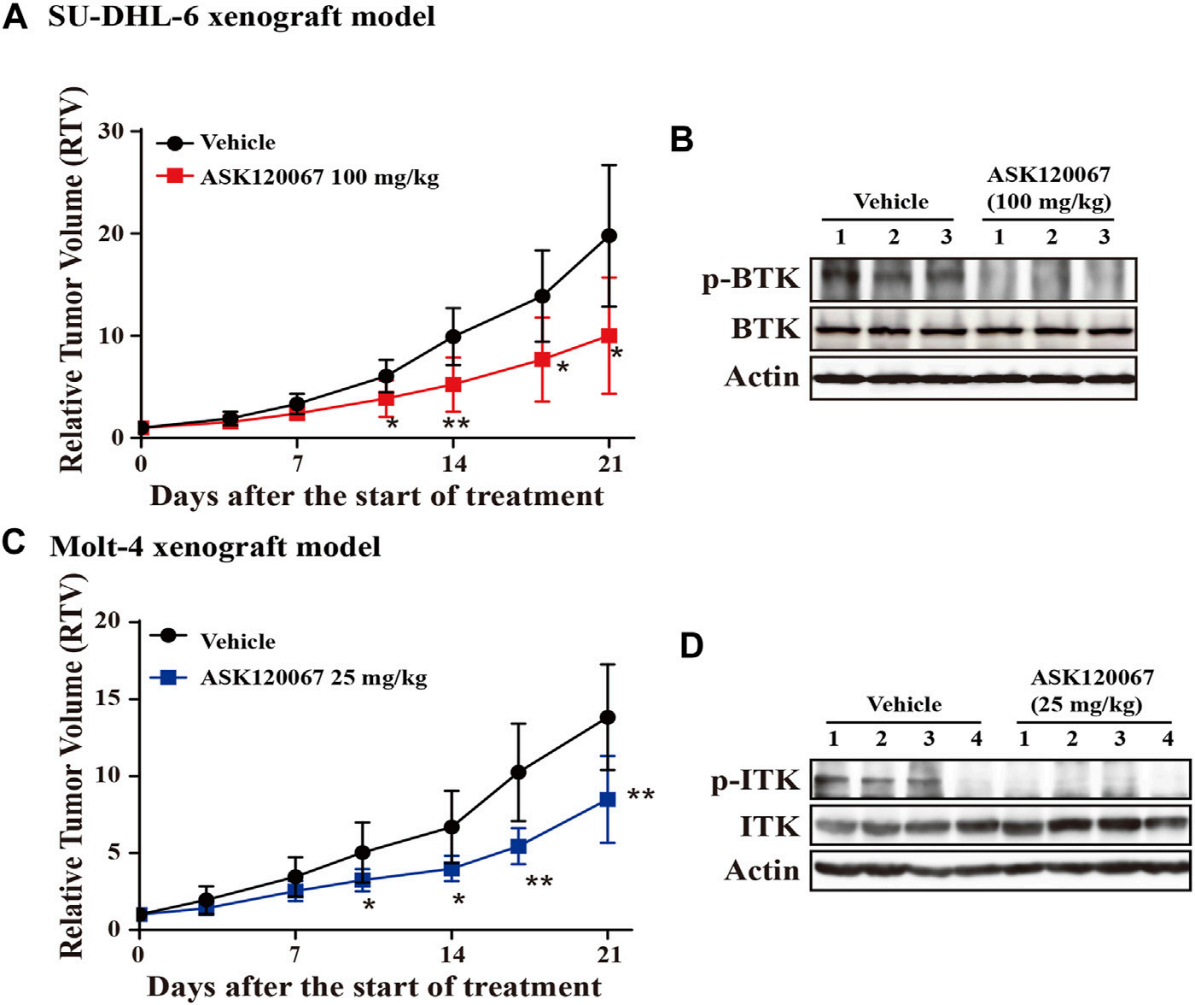
Oral administration of ASK120067 inhibited tumor growth in subcutaneous xenografts using SU-DHL-6 cells or Molt-4 cells. **(A)** Tumor-growth curve of a subcutaneous xenograft model in mice using SU-DHL-6 cells. Vehicle-group mice were treated with 1% Tween 80, and treatment-group mice were administered ASK120067 (100 mg/kg, p.o.) once a day for 21 days. Tumor size was measured twice a week. Data are the mean ± SD. The Student’s *t*-test was conducted to compare the relative tumor volume of ASK120067-treated tumors vs. vehicle-treated tumors (*p < 0.05; **p < 0.01). **(B)** Expression of p-BTK in tumor tissue after injection of SU-DHL-6 cells was evaluated by western blotting after 21 days of treatment. **(C)** Tumor growth of the subcutaneous xenograft model using Molt-4 cells was monitored after mice had been given vehicle or ASK120067 (25 mg/kg, p.o.) once a day for 21 days. Tumor size was measured twice a week. The Student’s *t*-test was conducted to compare the difference in ASK120067-treated tumors vs. vehicle-treated tumors. *p < 0.05; **p < 0.01. **(D)** Effects of 21 days of ASK120067 treatment on p-ITK expression in tumor tissue after injection of Molt-4 cells was evaluated by western blotting.

Administration of ASK120067 (100 mg/kg, p.o.) for 21 days led to significant tumor shrinkage in the xenograft model using SU-DHL-6 cells, with TGI = 57.50% (Figure 5A). Then, we conducted western blotting to measure p-BTK expression in tumor tissues. ASK120067 reduced the activation of BTK in tumor tissues (Figure 5B), which suggested a correlation between the anti-tumor potency and target-inhibition activity of ASK120067.

We also observed significant tumor shrinkage in the xenograft model using Molt-4 cells after administration of ASK120067 (25 mg/kg, p.o.) for 21 days, with TGI = 40.33% (Figure 5C). Consistently, expression of p-BTK in tumor tissues in mice in the 25-mg/kg group decreased 21 days after ASK120067 treatment (Figure 5D). Furthermore, ASK120067 treatment did not generate significant toxic effects and did not cause obvious body weight loss of mice.

These results showed that ASK120067 repressed the *in vivo* growth of B-cell lymphoma or T-cell leukemia by inhibiting hyperactivation of BTK or ITK, respectively.

## Discussion

In recent years, the development of targeted drugs based on the biological characteristics of tumors has provided more treatment options for cancer patients. BTK is a key kinase in the BCR signal transduction pathway. It has become a popular target for the treatment of B-cell malignancies.

The BTK inhibitor ibrutinib occupies an important position in lymphoma treatment because it brings a survival hope for patients with B-cell lymphoma, and promotes development of a chemotherapy-free era for such patients. ITK is important in signal transduction, influences the differentiation and development of T cells, and has a role in cytokine production. Therefore, ITK has become a promising target for the treatment of T-cell related diseases.

We demonstrated that the third-generation EGFR inhibitor ASK120067 (which is now a new drug application in China) inhibited BTK and ITK potently. Our preclinical model showed that ASK120067 was efficacious against B-cell/T-cell malignancy *in vivo* and *in vitro*. Also, the kinases irreversibly inhibitory activity of ASK120067 against BTK/ITK was similar with or even more effective potency than first-generation BTK inhibitor ibrutinib. Our research expands the clinical indications for ASK120067 and provides a new treatment option for B-cell lymphoma through targeting BTK. Our data could also provide: i) a new reference for the development of small-molecule inhibitors of ITK; ii) deeper insights into the clinical treatment of T-cell lymphoma by targeting ITK.

Primary and secondary resistance to ibrutinib have appeared in clinical practice, and the prognosis of patients with B-cell lymphoma is poor. Finding efficacious strategies to overcome drug resistance and control tumor progression is an urgent problem for lymphoma patients. The mechanism of action of ibrutinib resistance is complex. It has been reported that a mutation of myeloid differentiation primary response-88 in patients with Waldenström’s macroglobulinemia and abnormal activation of the phosphoinositide 3-kinase/protein kinase B signalling pathway can induce resistance to the effects of ibrutinib. ASK120067 used alone or combination with other drugs might be a strategy to overcome resistance (e.g., ibrutinib).

Based on the ibrutinib activity in blocking ITK kinase, the antitumor effect of ibrutinib against T-cell malignancies were examined in preclinical and clinical studies. According to a phase I clinical trial (NCT02309580) which focused on the treatment of ibrutinib for relapsed or refractory T-cell non-Hodgkin lymphoma patients, and a Phase II clinical study (NCT02581930) on patients who had metastatic melanoma with highly expressed ITK, ibrutinib did not achieved satisfied therapeutic effect. Further clinical researches against other T-cell lymphomas subtypes or combination of ibrutinib with other drugs need to be carried out (Kumar et al., 2018). Studies of ASK120067 for treatment of T-cell lymphoma *via* targeting ITK is also worth trying because ASK120067 is safe and well-tolerated in clinical trial against lung cancer.

## Conclusion

Our work demonstrated that ASK120067 exhibited significant anti-tumour activities against B-cell lymphoma and T-cell leukemia *in vivo* and *in vitro*, *via* targeting BTK and ITK. These data supported ASK120067 as a promising drug candidate for the treatment of B-cell or T-cell hematologic malignancies.

## Data Availability

The original contributions presented in the study are included in the article/supplementary material, further inquiries can be directed to the corresponding authors.
